# Isotropic non-white matter partial volume effects in constrained spherical deconvolution

**DOI:** 10.3389/fninf.2014.00028

**Published:** 2014-03-28

**Authors:** Timo Roine, Ben Jeurissen, Daniele Perrone, Jan Aelterman, Alexander Leemans, Wilfried Philips, Jan Sijbers

**Affiliations:** ^1^iMinds-Vision Lab, Department of Physics, University of AntwerpAntwerp, Belgium; ^2^Ghent University-iMinds/Image Processing and InterpretationGhent, Belgium; ^3^Image Sciences Institute, University Medical Center UtrechtUtrecht, Netherlands

**Keywords:** diffusion MRI, fiber orientation, partial volume effect, constrained spherical deconvolution, gray matter

## Abstract

Diffusion-weighted (DW) magnetic resonance imaging (MRI) is a non-invasive imaging method, which can be used to investigate neural tracts in the white matter (WM) of the brain. Significant partial volume effects (PVEs) are present in the DW signal due to relatively large voxel sizes. These PVEs can be caused by both non-WM tissue, such as gray matter (GM) and cerebrospinal fluid (CSF), and by multiple non-parallel WM fiber populations. High angular resolution diffusion imaging (HARDI) methods have been developed to correctly characterize complex WM fiber configurations, but to date, many of the HARDI methods do not account for non-WM PVEs. In this work, we investigated the isotropic PVEs caused by non-WM tissue in WM voxels on fiber orientations extracted with constrained spherical deconvolution (CSD). Experiments were performed on simulated and real DW-MRI data. In particular, simulations were performed to demonstrate the effects of varying the diffusion weightings, signal-to-noise ratios (SNRs), fiber configurations, and tissue fractions. Our results show that the presence of non-WM tissue signal causes a decrease in the precision of the detected fiber orientations and an increase in the detection of false peaks in CSD. We estimated 35–50% of WM voxels to be affected by non-WM PVEs. For HARDI sequences, which typically have a relatively high degree of diffusion weighting, these adverse effects are most pronounced in voxels with GM PVEs. The non-WM PVEs become severe with 50% GM volume for maximum spherical harmonics orders of 8 and below, and already with 25% GM volume for higher orders. In addition, a low diffusion weighting or SNR increases the effects. The non-WM PVEs may cause problems in connectomics, where reliable fiber tracking at the WM–GM interface is especially important. We suggest acquiring data with high diffusion-weighting 2500–3000 s/mm^2^, reasonable SNR (~30) and using lower SH orders in GM contaminated regions to minimize the non-WM PVEs in CSD.

## INTRODUCTION

Diffusion-weighted (DW) magnetic resonance imaging (MRI) is a non-invasive imaging method to investigate tissue microstructure via the measurement of the displacement of water molecules (Stejskal and Tanner, 1965; Jones, 2010). Diffusion in white matter (WM) neural tracts is anisotropic: it is larger parallel to the tract than in the perpendicular direction. In liquid, such as cerebrospinal fluid (CSF), diffusion is isotropic, i.e., equal in all directions. This diffusion property can be exploited to extract fiber orientations from DW data and investigate neural tracts in the brain WM using fiber tractography algorithms (Conturo et al., 1999; Basser et al., 2000; Mori and van Zijl, 2002; Jones, 2008; Tournier et al., 2010; Jeurissen et al., 2011).

The image resolution in DW-MRI is typically about 2–3 mm in all directions. Thus, significant partial volume effects (PVEs) are present in the measured signal (Alexander et al., 2001; Vos et al., 2011). These may be caused by multiple non-parallel neural tracts passing through a voxel (Vos et al., 2011; Jeurissen et al., 2013), or several tissue types present in a voxel (Pasternak et al., 2009; Metzler-Baddeley et al., 2012a).

Currently, the most common method in the analysis of DW-MRI data is diffusion tensor imaging (DTI; Basser et al., 1994a,b; Jones and Leemans, 2011; Tournier et al., 2011). The shortcoming of DTI is the inability to identify complex fiber configurations consisting of multiple fiber orientations (Alexander et al., 2001; Frank, 2001, 2002), present in 60–90% of WM voxels (Jeurissen et al., 2013). To overcome this, high angular resolution diffusion imaging (HARDI) methods (Tuch et al., 2002; Jansons and Alexander, 2003; Tournier et al., 2004, 2007; Tuch, 2004; Dell’Acqua et al., 2007; Descoteaux et al., 2007; Behrens et al., 2007) and methods based on diffusion spectrum imaging (DSI; Wedeen et al., 2005, 2008) have been developed. However, although able to identify complex fiber configurations, most of the HARDI methods do not account for PVEs caused by non-WM tissue, such as gray matter (GM) and CSF (Dell’Acqua et al., 2010; Metzler-Baddeley et al., 2012a).

The presence of non-WM PVEs is known in DW-MRI (Alexander et al., 2001; Pasternak et al., 2009; Dell’Acqua et al., 2010; Metzler-Baddeley et al., 2012a), but their effects in HARDI methods have not been widely studied. Diffusion in non-WM tissue is mostly isotropic within the resolution of DW-MRI (Dell’Acqua et al., 2010). Isotropic non-WM PVEs have been shown to affect DTI (Alexander et al., 2001; Pasternak et al., 2009) and tensor-derived measures (Metzler-Baddeley et al., 2012a). Pasternak and coworkers used constrained optimization of a bi-tensor model for “free water elimination” (FWE) in DTI (Pasternak et al., 2009), but they did not investigate GM PVEs. Metzler–Baddeley and coworkers used FWE to correct for CSF-contamination in tensor-derived measures in constrained spherical deconvolution (CSD) based tractography (Metzler-Baddeley et al., 2012a). Both fractional anisotropy (FA) and mean diffusivity (MD) were shown to increase in the presence of CSF-contamination (Pasternak et al., 2009). Moreover, diffusivity metrics were shown to be more sensitive to PVEs than anisotropy metrics (Metzler-Baddeley et al., 2012a). However, FWE-based approaches are not suitable for GM-contaminated regions.

In HARDI methods, very few studies account for the non-WM PVEs. The “ball and stick” model is the only method, which initially included an isotropic compartment and could be extended into multiple fiber orientations (Behrens et al., 2003, 2007; Jbabdi et al., 2012). In another study involving HARDI methods, isotropic PVEs were dampened by using adaptive regularization in the iterative Richardson–Lucy deconvolution algorithm (Dell’Acqua et al., 2010). Other methods that also account for isotropic compartments include diffusion basis spectrum imaging (Wang et al., 2011) and diffusion decomposition (Yeh et al., 2011; Yeh and Tseng, 2013). However, the non-WM PVEs are not taken into account and have not been studied earlier in CSD, one of the most popular, clinically feasible and readily available HARDI methods (Leemans et al., 2009; Tournier et al., 2012).

In this work, we perform simulations to assess non-WM PVEs in CSD (Tournier et al., 2004, 2007). This kind of comprehensive analysis has not been performed before, although the method is widely used and the consequences may be significant when studying the connectivity between GM regions. In addition, we analyze the proportion of voxels affected by isotropic PVEs, and present the fiber orientation distribution functions (fODFs) estimated with CSD in real data affected by non-WM PVEs.

## MATERIALS AND METHODS

We investigated the isotropic PVEs caused by non-WM tissue on fODFs estimated with CSD. DW signals were simulated with varying diffusion weightings, signal-to-noise ratios, fiber configurations, and tissue fractions. In addition, experiments with real data were performed.

### ESTIMATION OF FIBER ORIENTATIONS WITH CONSTRAINED SPHERICAL DECONVOLUTION

In CSD, the full fODF is deconvolved from the DW signal using a kernel constructed from the single-fiber response function (RF), which can be estimated from the data (Tournier et al., 2004; Tax et al., 2014). During the deconvolution procedure, constraints are imposed to suppress the negative peaks in the fODF (Tournier et al., 2007, 2008). The number of distinct gradient directions limits the maximum order of the spherical harmonics (SH) decomposition, which can be used in the estimation in the fODF. However, the constraints used to suppress the negative peaks in the fODF can be exploited to estimate higher order solutions and thus, describe more complex fODFs. This is called super-resolved CSD (Tournier et al., 2007).

To find the peaks of the fODF estimated with CSD, a Newton optimization algorithm was used to extract the local maxima of the fODF directly based on the SH decompositions (Jeurissen et al., 2013). Optimization was initialized on a dense set of uniformly distributed spherical sample points. A threshold of 33% of the maximum amplitude of the fODF was used to discard small peaks. A maximum of six of the highest peaks were identified. The peaks were clustered around the peaks of the average fODF calculated over all simulation repetitions performed with the same parameter configuration. Peaks further away than half of the crossing angle (with an upper limit of 35°) from any of the peaks of the average fODF were not included in the clusters. A mean dyadic tensor was then used to derive the mean orientation for each of the identified fiber clusters (Basser and Pajevic, 2000; Jones, 2003). This orientation was then compared to the true orientations of the fiber bundles. Peaks in clusters that were less than half of the crossing angle (with an upper limit of 35°) from the true orientations were defined as true, and rest of the peaks, also if they were not assigned to a cluster, as false. From the true clusters, accuracy and precision (95th percentile confidence interval, CI) with respect to the orientation of the mean dyadic tensor were calculated.

### SIMULATION OF THE DW SIGNAL WITH PVES

Two crossing WM fiber configurations were simulated with equal weights. The orientation of the first fiber bundle was randomly selected, after which the orientation of the second fiber bundle was calculated in spherical coordinates with the defined crossing angle. The resulting angle was verified to be correct in each case.

Then, the DW signal was simulated separately for different tissue types, and the resulting signals were combined assuming no exchange between the compartments (Leemans et al., 2005). The number of gradient directions uniformly distributed on the unit hemisphere was 64 (Jones et al., 1999). To eliminate any bias caused by the gradient orientations, a different gradient set was used for each simulated DW signal. Signal from the specific WM fiber configurations was combined with isotropic CSF and GM compartments. In addition, air compartments were simulated to investigate only the effect of reduced signal of the WM compartment without any isotropic diffusion. Derived based on Basser and Jones (2002), the combined simulated DW signal **S** is:

(1) $$S=\left(1-f_{isot}\right)\left(f_{fiber}e^{-Trace\left(bD_{fiber1}\right)}+\left(1-f_{fiber}\right)e^{-Trace\left(bD_{fiber2}\right)}\right)+f_{isot}e^{-Trace\left(bD_{isot}\right)},$$

where *f*_isot_ is the fraction of isotropic volume, *f*_fiber_ is the fraction of the first fiber compartment with respect to the WM compartment, **b** is the b-matrix summarizing the attenuation in all three directions of the diffusion tensor (including information of the diffusion-weighting and the gradient orientations; Mattiello et al., 1997), and **D**_fiber1_, **D**_fiber2_ and **D**_isot_ are the diffusion tensors of the two WM fibers and the isotropic compartment respectively (Basser et al., 1994b). The diffusion tensors were created with the following values. The MD for the simulation of different tissue types was 0.002 mm^2^/s for CSF, 0.0007 mm^2^/s for WM and GM (Dell’Acqua et al., 2010), and for air the signal was assumed to be zero. The FA was 0.8 for the WM signal and 0 for other tissue types. Rician noise was added to the combined DW signal. Finally, the DW signals were decomposed into an eighth-order series of SH (maximum possible order with the number of gradient orientations used).

### SIMULATION EXPERIMENTS

We performed simulation experiments to investigate the PVEs with different tissue compartments. Simulations and analyses of the simulation experiments were performed in Matlab (The MathWorks, Inc., Natick, MA, USA), by using dedicated software programmed by the authors.

The fraction of isotropic GM, CSF or air volume was varied from 0.00 to 0.95 with intervals of 0.05. We analyzed angles between the fiber populations in configurations ranging from 40° to 90° and with diffusion weightings (b-values) from 1000 to 3500 s/mm^2^. Signal-to-noise ratio (SNR) was calculated with respect to the non-diffusion weighted signal and simulated from 10 to 60, also generating a noiseless version of the DW signal. We performed 1000 repetitions with different noise realizations (resulting in Rician distributed data) for each parameter configuration. The fODFs were estimated from the simulated DW signals with CSD or super-resolved CSD using maximum orders of the SH from 4 to 14.

In addition to the isotropic volume fraction (VF) and PVE type (GM, CSF, or air) only one parameter at a time was investigated. The default values for the non-varying parameters were: *b*-value: 3000 s/mm^2^; angle between the crossing fiber configurations: 70°; SNR: 30. The default maximum order of the SH was 8 for CSD and 12 for super-resolved CSD.

### ACQUISITION AND ANALYSIS OF REAL DATA

High angular resolution DW data were acquired on a 3T MRI system with a 32-channel head coil. The subject gave written informed consent to participate in this study under a protocol approved by the local ethics committee. A single-shot echo-planar imaging (EPI) sequence was used with TR = 8100 ms, TE = 116 ms and 2.5 mm × 2.5 mm × 2.5 mm voxel size. The field of view (FOV) was 240 × 240 mm^2^ with a 96 × 96 acquisition matrix and the number of excitations (NEX) was 1. Fifty-four axial slices were imaged with 2.5 mm thickness and no gap. Diffusion sensitizing gradients with a *b*-value of 2800 s/mm^2^ were applied along 75 non-collinear directions. Ten images without diffusion weighting (*b* = 0 s/mm^2^) were acquired, of which one was acquired with reverse phase-encoding, for the purpose of EPI distortion correction. High-resolution anatomical T1-weighted images were acquired using a 3D magnetization-prepared rapid gradient-echo (MPRAGE) sequence (Mugler and Brookeman, 1990) with TR = 1900 ms, TE = 2.52 ms, TI = 900 ms and 1 mm × 1 mm × 1 mm voxel size (flip angle = 9° and NEX = 1). FOV was 250 mm × 250 mm × 176 mm with a 256 × 256 × 176 acquisition matrix.

The DW data were corrected for subject motion and eddy current induced distortions (Leemans and Jones, 2009; Andersson et al., 2012), and TOPUP was used to correct for EPI distortions (Andersson et al., 2003). The MRtrix package (J-D Tournier, Brain Research Institute, Melbourne, Australia, http://www.brain.org.au/software/; Tournier et al., 2012) was used for visualization of the real data. Tissue VFs for the DW data were estimated from the T1-weighted images, using a similar approach as presented by Smith et al. (2012).

The percentage of WM voxels affected by significant non-WM PVEs was estimated from real data. WM voxels were defined using a threshold of 25% WM tissue. The voxels with PVEs were estimated by using two threshold values: 25 and 10% non-WM volume.

## RESULTS

**FIGURE 1:**
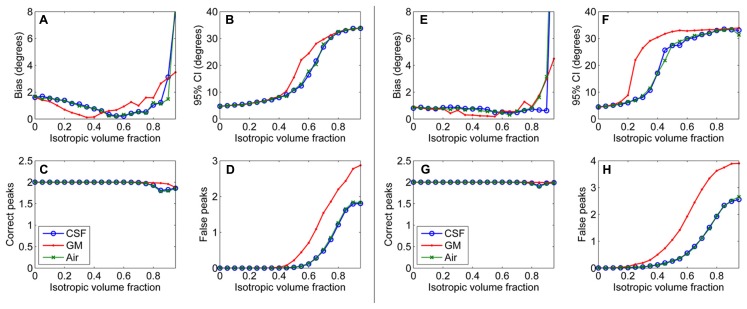
**Effect of isotropic volume fraction with CSF, GM, and air to bias, 95% confidence interval (CI), and the number of correct and false peaks estimated with CSD using up to eighth-order SH (A–D) and super-resolved CSD using up to 12th-order SH **(E–H**; with diffusion weighting 3000 s/mm^**2**^, angle 70°, and SNR 30)**.

First, results of the simulation experiments are presented. **Figure 1** shows the effects of isotropic non-WM VF in CSD (**Figures 1A–D**) and super-resolved CSD (**Figures 1E,H**). The bias and the 95% CI of the fiber orientations extracted with CSD are presented in **Figures 1A,B**. We also studied the effects on the number of correctly and falsely identified peaks (**Figures 1C,D**). The number of falsely identified peaks increased and the precision of the identified fiber orientations decreased, when the isotropic VF increased. The effects were stronger in GM than in CSF or air. However, the accuracy of the identified fiber orientations and the number of true peaks identified did not change until very high non-WM fractions. The similar performance with CSF and air PVEs using a high *b*-value indicates that the effect in CSF is mostly an SNR effect, which is clearly not the case in GM. Default values were used for the other parameters as specified in the methods section. The non-WM PVEs in super-resolved CSD, using up to 12th-order SH, started to affect the precision and the number of false peaks (**Figures 1F,H**) with lower fractions than when using up to eight-order SH. Accuracy remained high and was similar to the results when using SH up to eight-order. However, the ability to detect the two correct fiber orientations stayed higher with high isotropic fractions than while using SH up to eight order.

**FIGURE 2:**
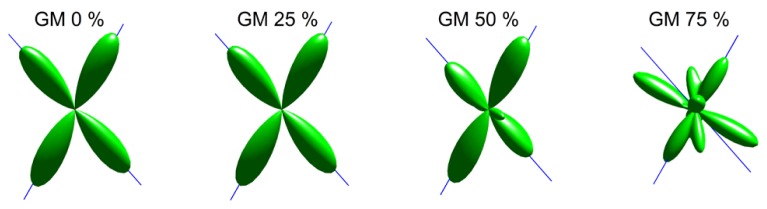
**An illustration of fODFs estimated with CSD with GM partial volume increasing from 0 to 75% (with up to eighth-order SH, diffusion weighting 3000 s/mm^**2**^, angle 70°, and SNR 30).** The blue lines correspond to the correct fiber orientations.

An illustration of the estimated fODFs based on only one noise realization per fraction is shown in **Figure 2**. The false peaks became more numerous and the correct peaks lost precision, when the isotropic VF increased. Next, the effects of varying maximum SH orders, diffusion weightings, SNRs, and angles between the two crossing fiber configurations were analyzed, while keeping the isotropic non-WM fraction constant at 0.5.

**FIGURE 3:**
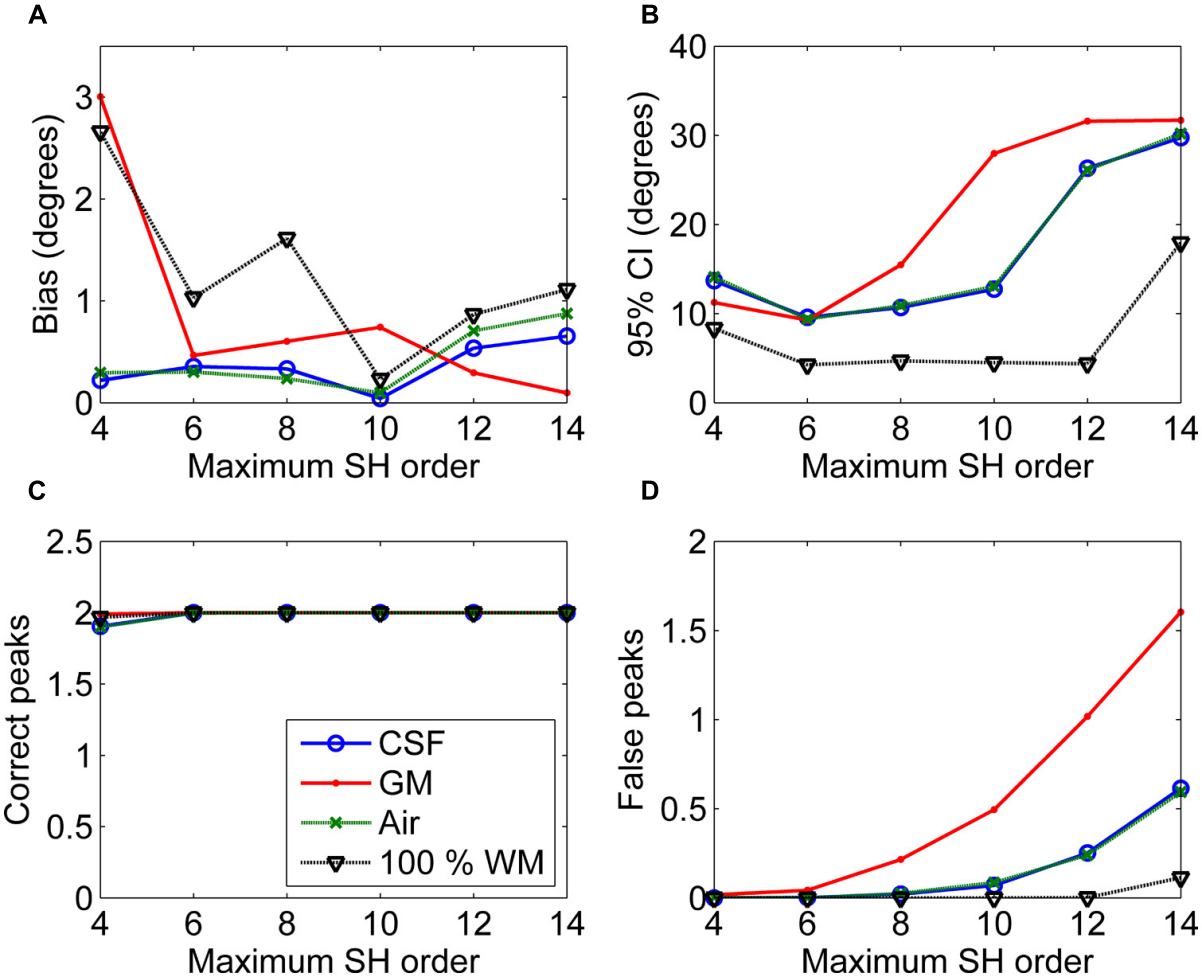
**The effect of varying maximum SH orders with 50% non-WM partial volume (CSF, GM, and air) to bias (A), 95% confidence interval (CI) **(B)**, and the number of correct **(C)** and false peaks **(D)** estimated with CSD (with diffusion weighting 3000 s/mm^**2**^, angle 70°, and SNR 30).** For comparison, 100% WM measures are provided.

In **Figure 3**, the effects of maximum SH order on the non-WM PVEs are shown. The 95% CI and the number of false peaks increased when higher maximum SH orders were used. Bias was low with all orders except for the lowest maximum order 4 with GM PVEs. However, the correct peaks could be found properly with GM PVEs even with the lowest order, but not with CSF or air PVEs.

**FIGURE 4:**
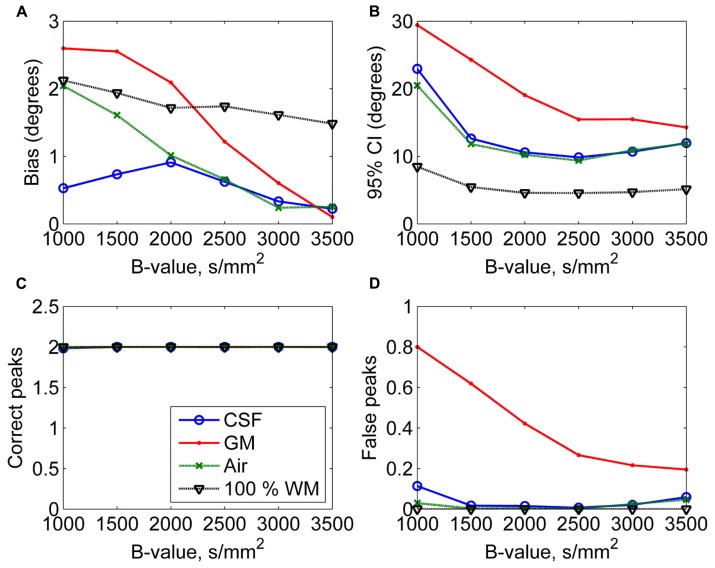
**The effect of varying diffusion weightings with 50% non-WM partial volume (CSF, GM, and air) to bias (A), 95% confidence interval (CI) (B), and the number of correct (C) and false peaks (D) estimated with CSD (with up to eighth-order SH, angle 70°, and SNR 30).** For comparison, 100% WM measures are provided.

In **Figure 4**, the effects of varying diffusion weightings to the non-WM PVEs are shown. The 95% CI and the number of false peaks increased when diffusion weighting decreased. The number of false peaks was high and the precision of the correct peaks was low under GM PVEs compared to CSF, air, or 100% WM regardless of the diffusion weighting. The difference between CSF and air PVEs was visible only with very low diffusion weightings of 1000–1500 s/mm^2^.

**FIGURE 5:**
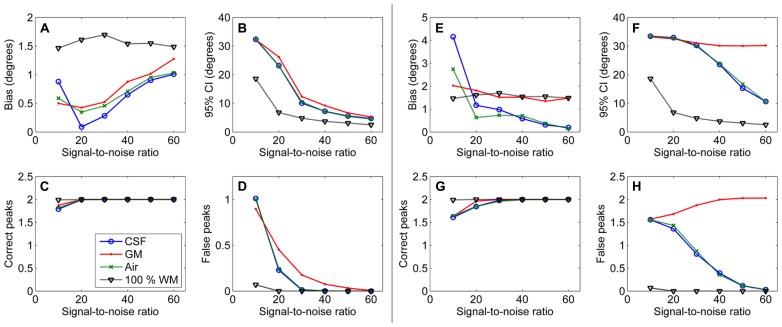
**The effect of varying SNRs with 50% (A–D) and 75% **(E–H)** non-WM partial volume (CSF, GM, and air) to bias, 95% confidence interval (CI), and the number of correct and false peaks estimated with CSD (with up to eighth-order SH, angle 70°, and diffusion weighting 3000 s/mm^2^).** For comparison, 100% WM measures are provided.

The effects of SNR on the non-WM PVEs are presented in **Figure 5**. **Figures 5A–D** show effects with 50% non-WM fractions and **Figures 5E–H** with 75% non-WM fractions. With 50% PVEs, increasing SNR improved the precision and reduced the number of false peaks identified. However, with 75% non-WM fractions, increasing SNR could not improve the situation with GM PVEs, and there were problems with precision also with high SNRs under CSF PVEs.

**FIGURE 6:**
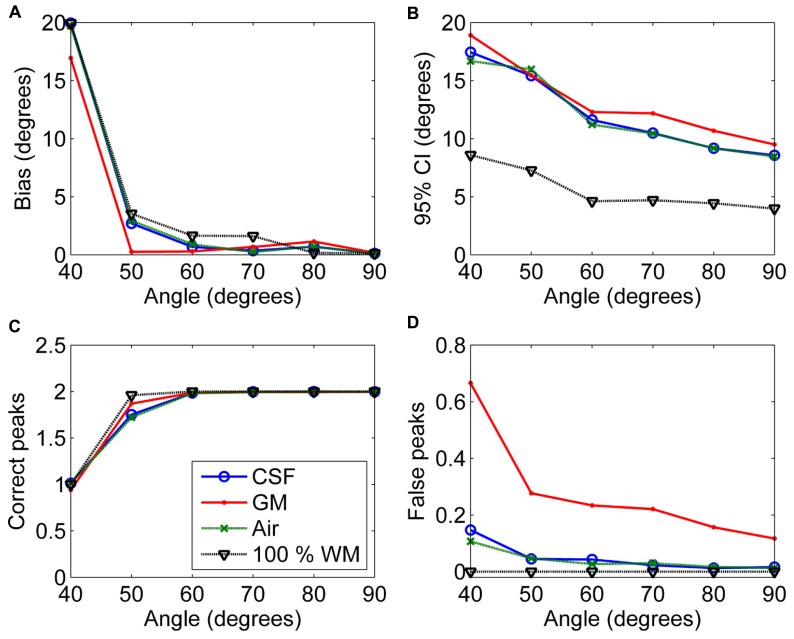
**The effect of varying angle between the two crossing fiber configurations with 50% non-WM partial volume (CSF, GM and air) to bias (A), 95% confidence interval (CI) (B), and the number of correct (C) and false peaks (D) estimated with CSD (with up to eighth-order SH, diffusion weighting 3000 s/mm^**2**^, and SNR 30).** For comparison, 100% WM measures are provided.

**Figure 6** shows the effects of varying angle between the two crossing fiber configurations. With an angle of 40° between the two fiber configurations, the correct peaks could not be properly identified. However, with an angle of 50°, they could still be reliably detected without isotropic PVEs, but any type of non-WM volume caused a decrease in the fraction of the correct peaks identified (**Figure 6C**). With higher angles, the correct peaks were identified correctly and without more bias than in pure WM (**Figure 6A**). The precision of the identified fiber orientations and the number of false peaks identified improved when the angle between the fiber configurations increased (**Figures 6B,D**).

From real data, we estimated that 35.7% of WM voxels, defined to have at least 25% WM volume, had significant PVEs with non-WM tissue, also defined to be more than 25% VF. Lowering the non-WM tissue threshold to 10%, the proportion of WM voxels affected by PVEs increased to 46.8%. Of these voxels with non-WM PVEs, 96.0% were affected by PVEs with GM and 5.3% with CSF.

**FIGURE 7:**
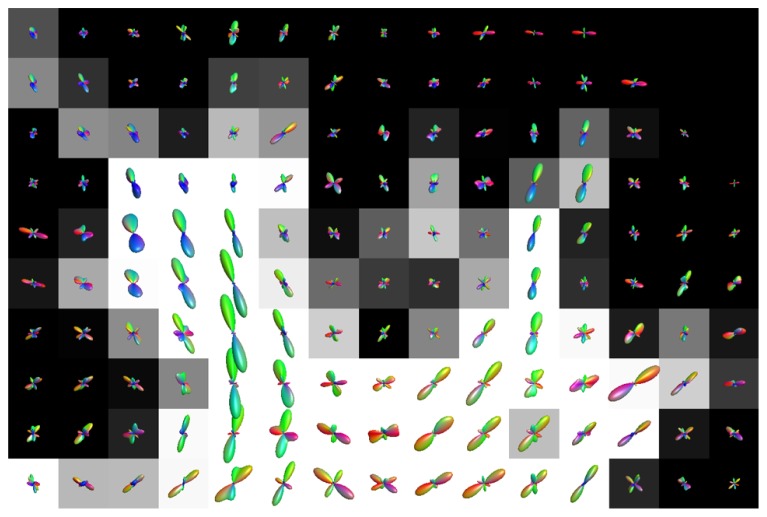
**Illustration of the effects of isotropic non-WM PVEs with GM in superior corona radiata with real data.** WM tissue probability map estimated from high-resolution anatomical MRI is visualized in the background, and fODFs estimated with CSD using up to eighth-order SH are overlaid.

**Figure 7** shows the fODFs estimated with CSD, using up to eight order SH, from real data overlaid on the WM tissue probability map of corona radiata extending towards cortical GM. The areas where WM interfaces with GM were affected both with CSD and super-resolved CSD. A large amount of voxels in the area had significant PVEs (gray-colored voxels), and perpendicular or spurious peaks appeared in the voxels with no apparent anatomical origin.

**FIGURE 8:**
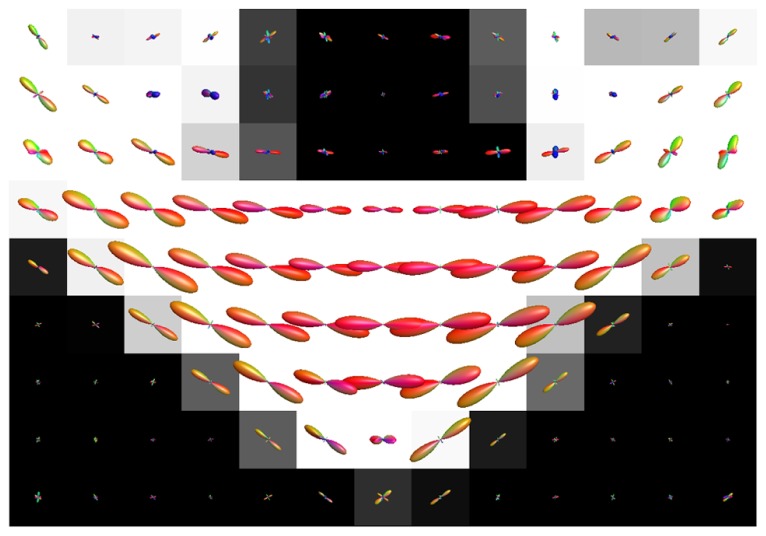
**Illustration of the effects of isotropic non-WM PVEs with CSF in corpus callosum with real data.** WM tissue probability map estimated from high-resolution anatomical MRI is visualized in the background, and fODFs estimated with CSD using up to eighth-order SH are overlaid.

**Figure 8** shows the effect of CSF PVEs on the estimation of fODFs at the interface between the corpus callosum and CSF. Spurious orientations can be noticed, but they are much smaller in amplitude and the principal fiber orientation can still be clearly distinguished.

## DISCUSSION

We studied the effects of isotropic non-WM partial volume on the fiber orientations estimated with CSD and super-resolved CSD by performing extensive simulations and real data experiments. CSD is a widely used method and knowledge about the implications of non-WM PVEs should be augmented.

Our results demonstrate that although CSD is efficient in the detection of PVEs caused by complex fiber configurations within a voxel, problems arise in the detection of the fODFs in the case of non-WM PVEs, which we estimated to be present in 35–50% of the WM voxels. As shown in **Figure 1**, the precision of the detected fiber orientations decreases and false peaks appear in the fODFs. This effect is most prominent for GM PVEs. The increase in bias with very high isotropic fractions may be at least partly caused by the inability to distinguish reliably both of the correct fiber orientations.

Part of these effects is due to the reduction of relative SNR in the WM tissue, which is caused by the decreased WM volume in the voxel, and not the isotropic diffusion properties of the non-WM tissue. PVEs with CSF tissue are mostly due to this effect, as shown by the similarity to air PVEs (**Figure 1**). Another part of the effects is caused by the isotropic diffusion, which invalidates the single fiber RF originally designed for pure WM. The more prominent PVEs in GM than in CSF or air are caused by this effect.

In addition, we showed that the PVEs increased when the maximum SH order increased. Therefore, the high maximum SH orders, although able to improve the angular resolution (Tournier et al., 2007), should be used with caution in the estimation of the fODFs under significant non-WM PVEs. Although the maximum angular frequency in the DW data is relatively low (Tournier et al., 2013), the fODFs contain higher angular frequencies, so a higher maximum SH order could still be useful in the estimation of the fODFs within pure WM regions. The use of lower diffusion weighting than generally used in HARDI sequences (i.e., less than 3000 s/mm^2^) increased the PVEs. Larger crossing angles could be detected with higher precision. With higher SNRs, moderate PVEs could be handled better, but high PVEs continued to decrease precision and increase the number of false peaks especially in GM.

Based on these results, we provide the following advice on how to operate CSD to maintain reasonable precision and number of false peaks under non-WM PVEs. Conditions with 95% CI lower than 20° and less than one identified false peak were considered reasonable. Thus, we suggest acquiring data with a high diffusion-weighting 2500–3000 s/mm^2^, and a reasonable SNR (~25–30). To extract the fiber orientations with CSD in regions with GM PVEs, we suggest using relatively low, from 6 to 8, maximum SH orders to minimize the loss in precision and the increase in the number of identified false fiber orientations. Nevertheless, the identified fiber orientations should be considered unreliable with higher than 60% GM and higher than 80% CSF VFs.

The isotropic PVEs, present in a significant proportion of WM voxels, lead to decreased precision and a high number of false peaks in the fODFs estimated with CSD, which in turn affects subsequent tractography algorithms, and may introduce false positives and hinder tract propagation into the cortex or near subcortical GM tissue. An algorithm already exists to discard tracts based on their anatomical feasibility and thus, only accept tracts that correctly propagate to the cortex (Smith et al., 2012). However, enabling the tracts to propagate properly into the cortex or adjacent to subcortical GM tissue would reduce the time needed for tracking and improve the precision of anatomically feasible tracts. Especially in connectomics, where reliable reconstruction of the fiber orientations profiles at the GM–WM interface is required to compute connectivity matrices, taking isotropic PVEs into account will be valuable.

Limitations of this study include the restriction to only one HARDI method, although it is one of the most commonly used ones (Metzler-Baddeley et al., 2012b; Emsell et al., 2013; Forde et al., 2013; Kristo et al., 2013; McGrath et al., 2013a,b; Reijmer et al., 2013a,b; Thompson et al., 2014). Previous studies indicate that the non-WM PVEs are present in DW-MRI in general (Alexander et al., 2001; Pasternak et al., 2009; Dell’Acqua et al., 2010; Metzler-Baddeley et al., 2012a). While some of the analysis methods already acknowledge or account for these PVEs (Behrens et al., 2003, 2007; Pasternak et al., 2009; Dell’Acqua et al., 2010; Wang et al., 2011; Yeh et al., 2011; Jbabdi et al., 2012; Yeh and Tseng, 2013), many of the currently used methods do not. For example, in CSD they have not yet been taken into account, and no detailed investigation about these effects had been performed previously. It is likely that also other methods which do not appropriately account for the non-WM PVEs will suffer from similar consequences. An additional limitation of this study is that there is no ground truth available in real data. Considering the clear effects demonstrated in the simulations, it is reasonable to assume that the spurious fiber orientations visible at the tissue interfaces are in fact false peaks also in real data. However, further experiments with real data are still necessary to completely understand the phenomenon and its effects in tractography. This would in turn help in the development of improvements for the fODF estimation with CSD, applicable also in real data, and thus allow improved tracking especially in the WM–GM interface.

In conclusion, we studied the effects of isotropic non-WM PVEs in CSD and found decreased precision and increased number of false peaks in the estimated fODFs. The effect was more pronounced with GM tissue. Considering the clear effects present in real and simulated data and the large proportion of WM voxels affected, it is important to take the non-WM PVEs into account in the extraction of fiber orientations with CSD. Therefore, we provide simple recommendations for the parameters used in the acquisition and the analysis, but acknowledge the need for more sophisticated methods to account for non-WM tissue in CSD.

## Conflict of Interest Statement

The authors declare that the research was conducted in the absence of any commercial or financial relationships that could be construed as a potential conflict of interest.
